# Human-Centered design requirements for clinical decision support in cardiac surgery perfusion

**DOI:** 10.1016/j.hfh.2026.100152

**Published:** 2026-08-17

**Authors:** Lakshmi Seelam, Sophie Yang, Letian Chen, Manisha Natarajan, Geoffrey Rance, Paul Ogara, Rithy Srey, Sanjana Mendu, Marco A. Zenati, Roger D. Dias, Matthew Gombolay

**Affiliations:** a Georgia Institute of Technology, Atlanta, GA, USA; b Cape Cod Healthcare, Hyannis, MA, USA; c VA Boston Healthcare System, Department of Veterans Affairs, Boston, MA, USA; d Invocirc Inc., Sudbury, MA 01776, USA; e Harvard Medical School, Boston, MA, USA; f Divisions of Cardiac Surgery and Emergency Medicine, Mass General Brigham, Boston, MA, USA

**Keywords:** Clinical decision support systems (CDSS), Perfusion, Human-centered design, Usability study

## Abstract

**Background:**

Perfusionists manage critical physiological parameters of cardiac surgery patients during cardiopulmonary bypass (CPB), requiring rapid, high-stakes decision-making under uncertainty. While artificial intelligence (AI) has the potential to enhance perfusionists’ decision-making and improve patient safety, there is limited understanding of how future AI-enabled clinical decision support systems (CDSS) should be designed to align with perfusionists’ workflows and cognitive demands.

**Objective:**

This study aims to identify human-centered design requirements for CDSS in cardiac perfusion and to conduct a formative evaluation of a prototype interface presenting simulated decision-support recommendations.

**Methods:**

A three-phase, multiple-method qualitative study was conducted with eight practicing perfusionists. Phase 1 involved semi-structured interviews to characterize workflow challenges and design requirements. Phase 2 engaged participants in structured prototype-feedback sessions to refine an interface concept for future AI-enabled decision support. Phase 3 assessed interface usability using scenario-based tasks with simulated AI recommendations designed by clinical experts and measured task success and exploratory self-reported perceptions.

**Results:**

Overall, perfusionists expressed positive perceptions of future AI-enabled CDSS concepts but emphasized the need to retain human control and receive contextually relevant explanations. Among three explanation formats evaluated, decision trees emerged as the most preferred explanation format across the study participants. In usability testing, participants achieved an 84% task success rate, reported positive perceptions of the prototype and perceived compatibility with perfusion workflows.

**Conclusion:**

This work contributes empirically grounded design requirements for future AI-enabled CDSS in safety-critical intraoperative settings. Key design considerations include: (1) prioritizing interpretable explanations (such as decision trees), (2) ensuring system recommendations align with perfusionists’ reported monitoring strategies, and (3) preserving clinician oversight through human-in-the-loop design frameworks.

## 1. Introduction

Artificial Intelligence (AI)-enabled clinical decision support systems (CDSS) have shown promise in enhancing clinical decision-making, particularly in settings involving interpretation of complex, time-sensitive data. (Chadebecq et al., 2023; Loftus et al., 2023) In cardiac surgery, AI has the potential to significantly impact the work of perfusionists who manage the cardiopulmonary bypass system (CPB), which serves as a substitute for the heart and lungs (Dias et al., 2021) in the operating room (OR). (Guyton et al., 2012; Wahr et al., 2013) Goal-directed perfusion (GDP) is a cognitively demanding task that requires continuous monitoring of physiological parameters and rapid adaptation to changing patient conditions. (Magruder et al., 2022) Even minor misjudgments in GDP can lead to severe patient complications or mortality, (Wiegmann et al., 2009; Trew et al., 2011; Mejak et al., 2000; Charrière et al., 2007) underscoring the need for advanced decision support tools designed around perfusionists’ information needs and clinical practices.

Clinical decision support systems (CDSS) are designed to assist clinicians by combining patient-specific data with clinical knowledge to generate alerts, recommendations, or reminders. Traditionally, CDSS have been implemented as rule-based systems, relying on predefined logic derived from clinical guidelines or expert knowledge. (Achour et al., 2001) While rule-based CDSS have demonstrated benefits in improving patient safety and adherence to clinical guidelines, such approaches remain limited in their ability to adapt to complex, dynamic patient conditions and may contribute to alert fatigue and workflow disruption. (Sutton et al., 2020)

Recent advances in AI and machine learning (ML) have expanded the capabilities of CDSS by enabling data-driven modeling of complex clinical patterns and large-scale patient data. AI-enabled CDSS can generate context-aware recommendations that are difficult to encode using rule-based logic. For instance, AI models could be trained to offer recommendations for fine-grained adjustments to the CPB system during cardiac perfusion from real-world expert data, which is difficult to encode manually. (Dias et al., 2022; Natarajan et al., 2025) However, successful deployment of CDSS depends not only on analytical performance but also on transparency, usability, and alignment with clinical workflows. Black-box models can limit clinicians’ ability to interpret system outputs and appropriately calibrate trust, which can hinder adoption. (Shortliffe & Sepúlveda, 2018)

Designing AI-enabled CDSS for clinical practice requires moving beyond technical considerations such as data quality, model performance, and fairness to account for human factors. Clinicians differ in expertise, familiarity with AI, and decision-making practices, all of which influence how they interpret and rely on system outputs. (Knop et al., 2022) Supporting meaningful human-AI interaction without increasing cognitive burden is especially challenging in high-stress clinical environments such as cardiac surgery, where user attention is limited and decisions are time-critical. (Shortliffe & Sepúlveda, 2018).

A shift toward human-centered AI design is also reflected in the Healthcare 5.0 paradigm, which emphasizes technologies that augment human capabilities through adaptive and context-aware human–machine collaboration, including AI copilots and intelligent decision support tools. (Gomathi et al., 2023) Within this paradigm, explainable artificial intelligence (XAI) has emerged as a key approach for improving transparency and fostering user trust. Prior work has examined how explanations influence user understanding and decision-making in AI-enabled systems. For instance, Gombolay et al. explored the effects of XAI on user reliance when presenting AI reasoning to domain experts in diagnosing neurological disorders. (Gombolay et al., 2024) Similarly, Panigutti et al. conducted a user study on an AI-based clinical decision support system, focusing on the impact of explanations as well as how they are perceived. (Panigutti et al., 2022) Although studies in healthcare AI have investigated explainable models and clinician trust, these efforts have largely focused on diagnostic or postoperative contexts, with limited attention to intraoperative, time-critical environments.

Moreover, explanations alone do not guarantee effective human–AI interaction. The usefulness of explanations is heavily context-dependent, as users’ informational needs vary based on task demands, expertise, and situational constraints. (Liao et al., 2020, April) Even technically accurate explanations may fail to align with users’ cognitive processes or support effective decision-making, which emphasizes the need for nuanced research into the objective and subjective facets of XAI. (Kenny et al., 2021).

Beyond XAI, human factors research provides additional insight into challenges associated with integrating AI into clinical workflows. Prior research highlights that some of the main factors affecting the acceptance of CDSS by physicians is the ease of use and trust in the system. (Bates et al., 2003; Shibl et al., 2013) Situation awareness (SA) is critical in dynamic, high-risk environments such as the OR, where clinicians must continuously perceive, interpret, and anticipate changes in a patient’s condition. (Endsley, 2017; Dias et al., 2022) However, the introduction of automation tools such as CDSS does not inherently improve SA. Poorly designed systems may increase cognitive workload, obscure important system dynamics, or direct attention toward limited subsets of information (Endsley & Jones, 2025), thereby impairing clinicians’ understanding of the patient state. Further, integrating automation in clinical workflows also introduces challenges related to trust and reliance, as clinicians may over-rely on low-quality system recommendations (misuse) or under-utilize high-quality recommendations (disuse), depending on how system outputs are presented. (Lee & See, 2004) Both failure modes can degrade performance and increase cognitive workload. (Parasuraman & Riley, 1997) Therefore, effective CDSS design requires not only accurate predictions but also interfaces that support situation awareness, present interpretable and temporally relevant information to elicit appropriate reliance from clinicians using the interface.

In safety-critical domains, recent work has further emphasized that usability alone is insufficient for evaluating AI-enabled systems, as effective human–AI collaboration depends on how joint human-AI decision-making unfolds under real-world constraints, and the ability of users to recognize and recover from system limitations. (Rayo, 2017; Morey et al., 2023; Morey et al., 2025) Designing decision support tools for such environments therefore requires moving beyond traditional usability metrics to consider how system behavior, explanations, and interaction design jointly shape decision-making in practice. Despite growing interest in AI-enabled CDSS, there remains a lack of human-centered design research examining the requirements and interface preferences of such systems to support real-time decision--making in safety-critical intraoperative environments. In particular, limited work has examined the design requirements for future AI-enabled CDSS in perfusion workflows during CPB, where decisions are time-sensitive, and errors can have immediate consequences for patient outcomes.

To address this gap, this work presents a human-centered design approach to identify requirements for future AI-enabled CDSS in cardiac perfusion. We followed a qualitative Human-Computer Interaction (HCI) research process to study and evaluate how prospective decision-support interfaces could be best designed around the specific needs of perfusionists. Our approach consists of a three-phase design to (1) characterize the pain points of perfusionists, (2) obtain structured feedback on interface concepts and simulated recommendation explanations, and (3) conduct usability testing of a prototype interface to inform design guidelines for interventions. We aim to *identify design requirements and interface preferences that can guide the future development and evaluation of AI-enabled CDSS to support expert decision-making in high-stakes intraoperative environments, such as cardiac perfusion.*

## 2. Problem statement

Perfusionists manage critical physiological parameters during CPB while accessing information distributed across multiple monitors and records and coordinating with other members in the OR for long durations. These workflow demands motivate the design of future AI-enabled clinical decision support systems that align with perfusionists’ monitoring practices and present recommendations with appropriate explanations. In this work, we therefore aim to address the critical design aspects of future AI-enabled CDSS in perfusion, including **what** information should be displayed and **how** recommendations and accompanying explanations should be presented to support perfusionists’ decision-making.

## 3. Materials and methods

In this section, we describe our study design and procedures. This study employed a three-phase, multiple-method qualitative design to investigate how AI-enabled CDSS can be effectively designed for cardiac perfusion. The study consisted of (1) exploratory interviews to understand the requirements for designing CDSS to support perfusionists, (2) structured prototype-feedback sessions to evaluate a low-fidelity interface and obtain feedback from perfusionists, and (3) usability testing to evaluate the final prototype. The three-phase iterative approach enabled the identification of user needs, development of interface concepts, and evaluation of user interaction with a prototype system.

Importantly, this study did not involve the development or deployment of a real-time AI model. Instead, the recommendations and explanations were simulated and manually constructed by domain experts on the research team using historical surgical data and clinical expertise. This approach allowed systematic evaluation of interaction design, user preferences, and interface requirements for AI-enabled decision support without confounding effects of model performance.

The study design was informed by participatory design principles, which emphasize involving end users in identifying needs and shaping design outcomes to promote alignment with real-world practices. (Sanders & Stappers, 2008; Spinuzzi, 2005) Specifically, in healthcare settings, participatory design approaches have been shown to facilitate integration of multiple clinical perspectives and improve the usability and relevance of health information technologies. (Hose et al., 2023; Panigutti et al., 2023) In this work, we employ participatory design in the form of interviews and structured prototype-feedback sessions to identify requirements and refine interface concepts for future AI-enabled CDSS in perfusion.

The reporting of the qualitative components of this study was informed by the Consolidated Criteria for Reporting Qualitative Research (COREQ) to promote transparency in participant recruitment, data collection, researcher roles, and analysis. (Booth et al., 2014).

### 3.1. Participants

Eight practicing perfusionists (age range: 27 – 59 years) were recruited across the three study phases using convenience sampling through professional networks and social media outreach. Perfusionists represent a highly specialized clinical population, which limits the available participant pool. Given the qualitative and exploratory aims of this work, the small sample size is consistent with prior work demonstrating that thematic saturation in qualitative interviews is often achieved with small samples. (Guest et al., 2006) The goal of recruitment was to capture rich, context-specific insights that can inform the design of effective AI-enabled CDSS, rather than achieve statistical generalizability.

Each study session lasted approximately 60 minutes per participant. The study was approved by the Institutional Review Board (IRB) at the primary researchers’ institution. The participants did not receive compensation, which may introduce selection bias toward individuals with an intrinsic interest in research or a favorable disposition toward AI-enabled technologies, thereby limiting the generalizability of the findings. All perfusionists recruited in our study have received training through either undergraduate or master’s programs. See Fig. 2 for a detailed breakdown of demographic information.

### 3.2. Phase 1: exploratory interview

Phase 1 consisted of semi-structured interviews designed to characterize perfusionists’ workflows, decision-making processes, information needs, and opportunities for CDSS in cardiac surgery. Six perfusionists participated in one-on-one interviews conducted via video conferencing.

Semi-structured interviews were selected to balance consistency across participants with flexibility to explore individual experiences in depth, which is particularly appropriate for investigating complex clinical practices. (Booth et al., 2014) An interview guide was developed to ensure coverage of key topics while allowing participants to elaborate on their experiences. Interview questions focused on: (1) roles and responsibilities of perfusionists during surgery, (2) existing decision-making aids and their limitations, (3) definitions of successful and non-ideal surgical outcomes, (4) workflow challenges and pain points, and (5) perceptions of how future CDSS could support performance.

All interviews were audio-recorded and supplemented with detailed researcher notes. Audio recordings and notes served as the primary data sources, consistent with qualitative research best practices. (Booth et al., 2014).

#### 3.2.1. User story development

User stories were developed to represent perfusionists’ workflows across preoperative, intraoperative, and postoperative phases. Participants were asked to describe their tasks and decision-making processes in detail, and these descriptions were synthesized into structured narratives, capturing key decision points and information requirements.

As perfusion practices can vary across hospitals and facilities, we aggregated the workflow steps across participants to develop a generalized representation of tasks. This process enabled systematic characterization of task complexity and identification of interaction points where future CDSS can provide value. Two members of the research team with HCI training independently reviewed the interview recordings and notes to construct user stories. The resulting user stories were iteratively refined through discussion until consensus was reached. The user stories provided a structured representation of workflow tasks, decision points, and interface needs relevant to subsequent prototype development. Supplemental 1 depicts our user stories.

#### 3.2.2. Inductive qualitative synthesis using affinity mapping

Affinity mapping was used as part of an inductive qualitative synthesis to organize the interview data and identify recurring patterns across participants. Interview excerpts and researcher notes were grouped based on conceptual similarity and iteratively organized into higher-level categories. This approach follows established contextual design methods, in which affinity diagrams have been used to synthesize qualitative data by clustering related observations and revealing relationships across user experiences. (Beyer & Holtzblatt, 1999; Gao et al., 2024) Two researchers with training in HCI independently reviewed and grouped the data, and the categories were refined through iterative discussion until consensus was reached.

### 3.3. Phase 2: participatory prototype-feedback sessions

Phase 2 consisted of participatory prototype-feedback sessions aimed at collaboratively developing and refining interface concepts for AI-enabled CDSS. Four perfusionists participated in this phase, including participants from Phase 1 to maintain continuity of domain knowledge.

Consistent with participatory design methodology, prototype-feedback sessions were structured to enable collaboration between researchers and domain experts, allowing participants to contribute experiential knowledge and shape system design through iterative feedback. (Sanders & Stappers, 2008)

#### 3.3.1. Prototype-Feedback procedure

Each session consisted of three components: (1) a demographic survey, (2) an interactive prototype-feedback activity, and (3) a semi-structured follow-up interview.

Participants were presented with preliminary design concepts in the form of low-fidelity wireframes, informed by Phase 1 findings. The design concepts were intentionally abstracted to avoid constraining design exploration and to encourage participants to critique, refine, and extend the features of the proposed system. During the session, discussion prompts were designed to elicit perfusionist perspectives on: (1) preferred modes of interaction with the CDSS, (2) timing and presentation methods for recommendations, (3) expectations for explanations from CDSS, and (4) compatibility within existing clinical workflows. All sessions were audio-recorded and supplemented with researcher notes.

#### 3.3.2. Interface prototyping

Low-fidelity wireframes were developed to facilitate discussion and iterative refinement of interface concepts. Wireframes are simple visual blueprints used as a bridge for communication between researchers, designers, and participants that are commonly used in participatory design and enable rapid exploration of design alternatives. (Shneiderman et al., 2016) Our wireframes were composed of three primary components: (1) a decision support panel for system-generated recommendations, (2) a patient information display, and (3) a live surgical event feed. The interface also included placeholder regions for patient-related information (e.g., medications, surgical history, demographics). These fields were intentionally left unspecified to allow the participants to indicate which information they considered most relevant. Participants interacted with the wireframes and provided feedback on layout, functionality, and information prioritization.

#### 3.3.3. Scenario-Based design

To support the evaluation of user interaction with decision support features, clinical scenarios were developed in collaboration with domain experts, including perfusionists and cardiac surgeons on the research team. Each scenario included a text description of the surgical case and a recommendation from a hypothetical AI system to simulate the interaction with the perfusionist. Three explanation formats were incorporated to explore different approaches to presenting decision rationale: (1) a decision tree, (2) a probability score table, and (3) a counterfactual example. Decision tree explanations were presented as graphical flowcharts with Yes/No checks leading to a recommended action. Probability-based explanations displayed likelihood estimates associated with alternative actions (to mimic the hypothetical AI model’s confidence in choosing different actions). For the counterfactual explanation, natural language describing how the scenario should change for the alternative action to be appropriate is provided. The simulated scenarios were used to ground prototype-feedback discussions in realistic clinical contexts and to elicit feedback on how recommendations and explanations should be presented. Following the interaction activity, perfusionists participated in a semi-structured interview to provide feedback on the overall interface, including perceived usefulness, missing functionality, and suggested improvements.

#### 3.3.4. Data collection and analysis

Data collected during the prototype-feedback sessions included audio recordings, participant feedback during interactive activities, and responses from follow-up interviews. Qualitative data were analyzed using an inductive approach, consistent with Phase 1 analysis, to identify patterns in participant preferences and design considerations. Two members of the research team with training in HCI independently reviewed the data and collaboratively developed patterns and design considerations through iterative discussion.

### 3.4. Phase 3: usability testing

**Fig. 1.**
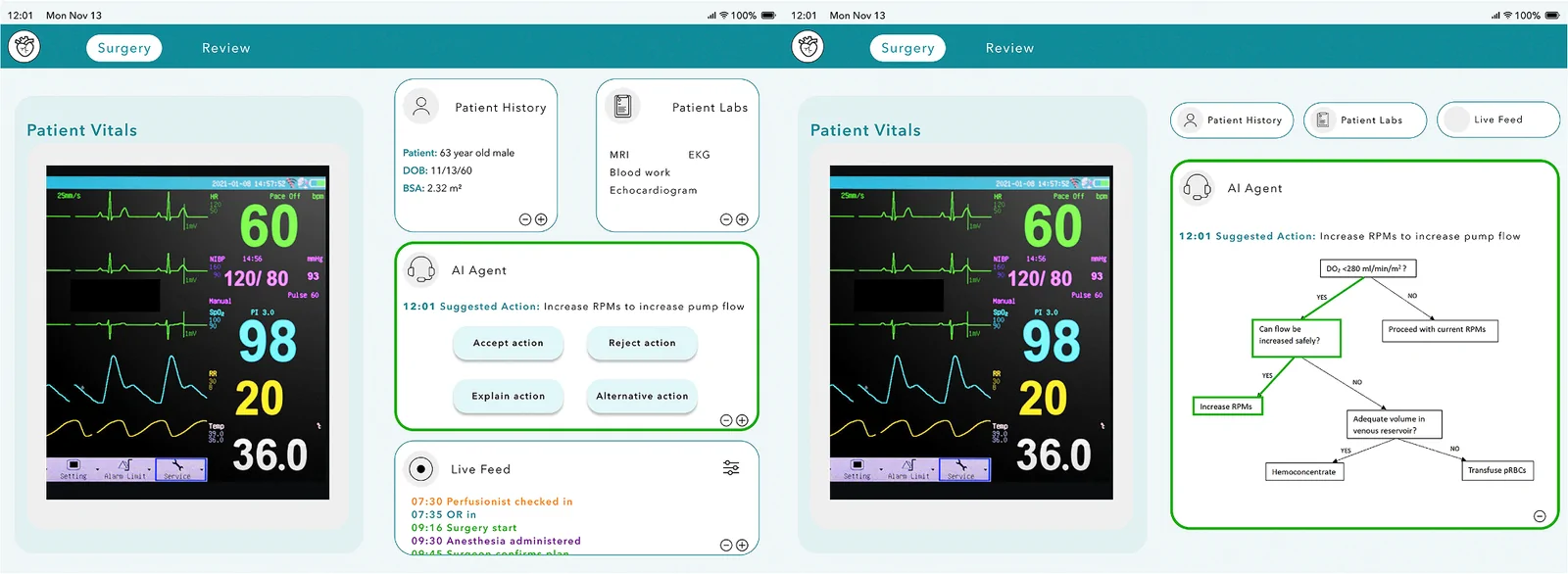
We present a three-phase formative design study examining requirements and interface preferences for prospective Artificial Intelligence (AI) decision-aids in cardiovascular perfusion. We began by conducting exploratory interviews and prototype-feedback sessions to understand and document design requirements. Then, we created a Graphical User Interface (GUI) based on design requirements and conducted usability testing on the GUI. The figure above is our final GUI design. On the left, the simulated AI agent provided a suggestion based on patient data. On the right, the simulated AI agent explained its suggestion using a decision tree, with the reasoning path highlighted in green. In the figure, DO2 represents Oxygen Delivery, RPMs represent Revolutions per minute, pRBCs represent Packed Red Blood Cells.

Phase 3 consisted of usability testing of a high-fidelity interactive prototype developed based on findings from Phases 1 and 2. An overview of the final interface is shown in Fig. 1, including simulated decision-support recommendations, patient information displays, and explanation visualizations. Five perfusionists participated in the usability testing phase.

The objective of this phase was to evaluate interface usability and perceived utility of the prototype in supporting perfusionists’ decision-making. Specifically, the evaluation focused on: (1) alignment with perfusionists’ expectations, (2) ease of use and intuitiveness of interface, (3) perceived compatibility within a highly demanding surgical environment, (4) user perceptions of interacting with simulated AI recommendations, and (5) opportunities for further design refinement.

Usability testing consisted of two parts. The first was a task-based evaluation approach combined with a think-aloud protocol, a widely used method in human–computer interaction for capturing user reasoning and interaction challenges during system use. (McDonald et al., 2012) Participants were presented with the prototype and asked to verbalize their thought processes while completing a list of tasks, enabling observation of both verbal and behavioral responses. Following the task-based evaluation, participants completed a semi-structured interview to provide qualitative feedback on usability and overall prototype experience.

#### 3.4.1. Task-based evaluation

Perfusionists were asked to complete fifteen tasks (see Supplemental 2) designed to cover all major functionalities of the interface. Tasks included locating key patient-specific information (e.g., imaging data), interpreting simulated AI recommendations, and requesting explanations for the presented recommendation.

Tasks were performed within two simulated surgical scenarios (see Section 3.3.3) to provide contextual grounding. Each scenario included simulated recommendations and the corresponding explanation in the form of decision trees (as informed by Phase 2 findings). Task performance was evaluated based on whether participants were able to complete each task independently without assistance.

#### 3.4.2. Measures

Usability evaluation included both objective and subjective measures. Objective performance was assessed using the task completion rate, defined as the proportion of tasks successfully completed. Subjective perceptions were captured using a brief exploratory self-report survey assessing anticipated emotional responses to system use. The survey included five items: “I would feel comfortable,” “distracted,” “anxious,” “relaxed,” and “annoyed.” Participants rated each item on a 5-point Likert scale ranging from 1 (Not likely at all) to 5 (Very likely).

Two members of the research team with training in HCI reviewed the qualitative data from the think-aloud protocols and follow-up interviews to identify usability challenges, user preferences, and opportunities for improvement.

#### 3.4.3. Limitations of usability evaluations

While usability testing provides insights into interface design and user interaction, prior work has shown that usability evaluations alone are insufficient to fully assess human-AI collaboration in safety-critical environments. (Rayo, 2017; Morey et al., 2023; Morey et al., 2025) In particular, such evaluations do not capture how users adapt to system errors, how reliance evolves over time, or how joint human-AI decision-making unfolds under real-world constraints. These limitations are important when interpreting the findings of this study, as the evaluation focuses on interaction with a simulated CDSS rather than performance in real clinical settings. We further elaborate on the limitations of this work in Section 5.5.

## 4. Results

In this section, we highlight the key findings from each phase of the study and elaborate on how these findings shaped the subsequent design choices for later phases in the study. An overview of how findings from each phase informed subsequent prototype refinements is presented in Table 1.

### 4.1. Phase 1: workflow challenges and design requirements

Exploratory interviews identified several challenges in perfusion practice and highlighted areas where future CDSS may provide value. Through user stories and affinity mapping, we synthesized key patterns and insights across user feedback:

- Fatigue – Fatigue is perceived as the primary cause of surgical errors. Participants described fatigue as arising from sustained vigilance demands, including continuously scanning monitors and responding to multiple alarms during long procedures.
- Communication – Participants reported that effective, closed-loop communication in the OR can be compromised due to overcrowding, competing conversations, and alarms.
- Better Charting – Participants indicated that existing charting systems were inefficient, often requiring retrospective documentation post-surgery, and limiting access to relevant patient information during surgery, when it is most actionable.
- Role of AI in CDSS - Participants expressed interest in AI-enabled tools that could support real-time monitoring, highlight relevant information, and provide reminders. CDSS were generally described as supplementary tools that could support ongoing decision-making while preserving clinician control.
- Lack of innovation – Aside from physical equipment upgrades, participants indicated that perfusion as a practice has experienced limited technological innovation for almost 20 years.

#### Key Takeaways:

Findings from Phase 1 suggest that future CDSS should highlight relevant information, support awareness of clinically important events in real-time, and function as supplementary aids that assist clinicians without disrupting existing workflows. These findings informed the development of the low-fidelity wireframe interface used to evaluate during the prototype-feedback sessions (Phase 2).

### 4.2. Phase 2: prototype-feedback takeaways and design revisions

The prototype-feedback sessions provided additional insights into preferred interaction patterns, explanation formats, and perceived compatibility of CDSS concepts with perfusion workflows. Based on participant feedback, we implemented further design revisions for Phase 3 usability testing (Section 3.4).

#### 4.2.1. Perceptions of AI-enabled decision tools

Perfusionists had largely positive reactions to the concept of an AI agent providing recommendations. Participants viewed the AI as a “second pair of eyes” that would alert them to any events that necessitated intervention. However, perfusionists wanted to be able to inspect the inner workings of the AI agent (e.g., through XAI) to boost transparency. At the same time, participants emphasized the importance of maintaining control, avoiding mandatory interaction with AI suggestions as they may not always have the time to respond. Key quotes exemplifying this feedback:

- “If AI can anticipate something faster than we might notice, that would be nice”
- “Would face a lot of resistance if it’s something you have to do; I kinda like a timeout”

Participants also provided direct feedback regarding the wireframe interface as they expressed confusion regarding the function of “reject action” and the function to request a “different action”, which was revised for the subsequent phases of the study.

##### Key Takeaways:

Findings indicate that participants preferred decision support systems that provide optional and non-intrusive recommendations while preserving clinician control.

#### 4.2.2. Preference for explanation formats

In Phase 2, we compared three explanation formats accompanying simulated AI recommendations to the perfusionists. Participants preferred the decision tree format over the probability chart and the counterfactual example. Representative feedback from the Phase 2 semi-structured interviews include:

- “It allows me to construct my own decision making as well”
- “Explanations will help me understand the AI’s mental model, both during surgery and after surgery as a debriefing tool”

**Fig. 2.**
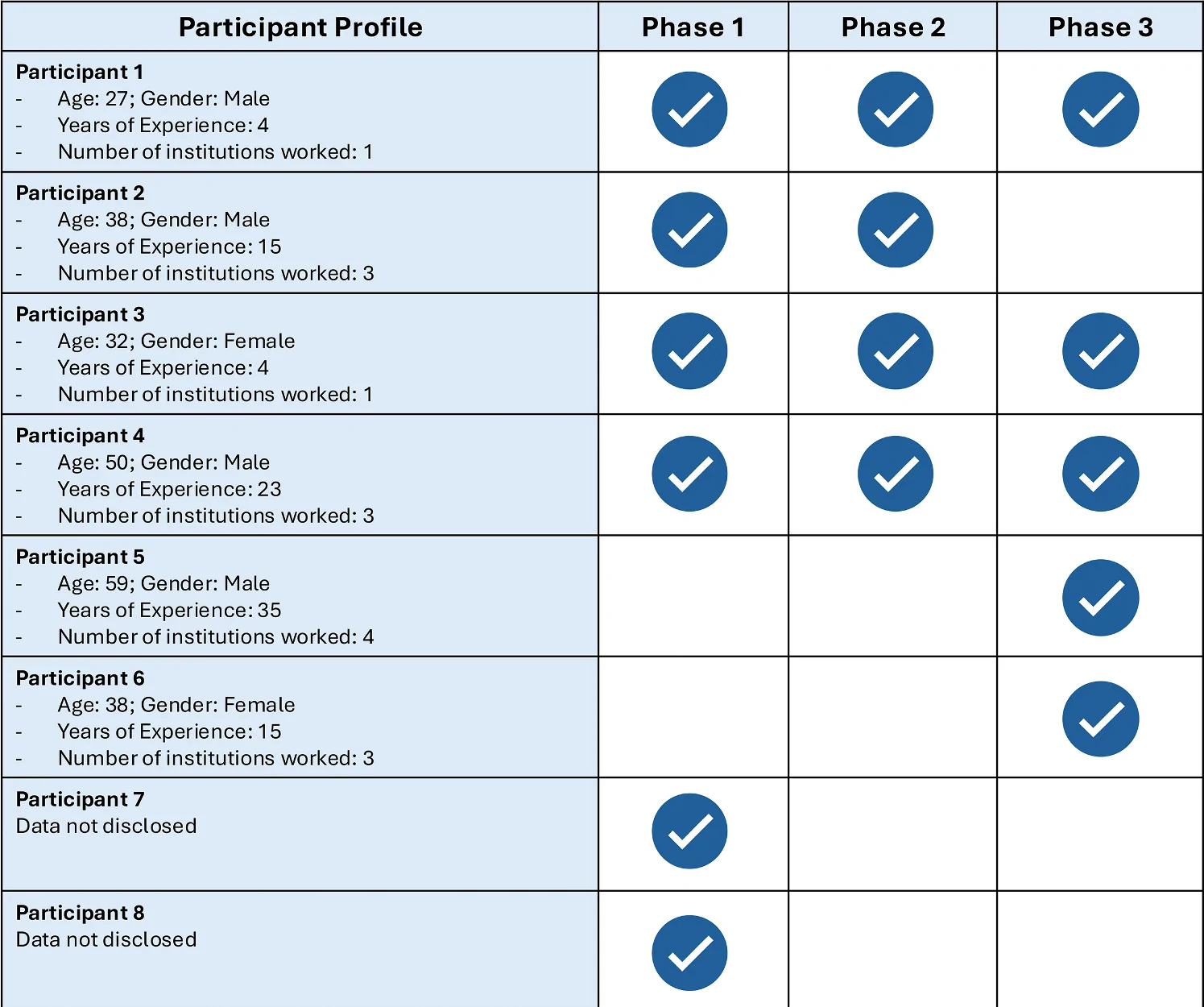
Participant chart.

**Fig. 3.**
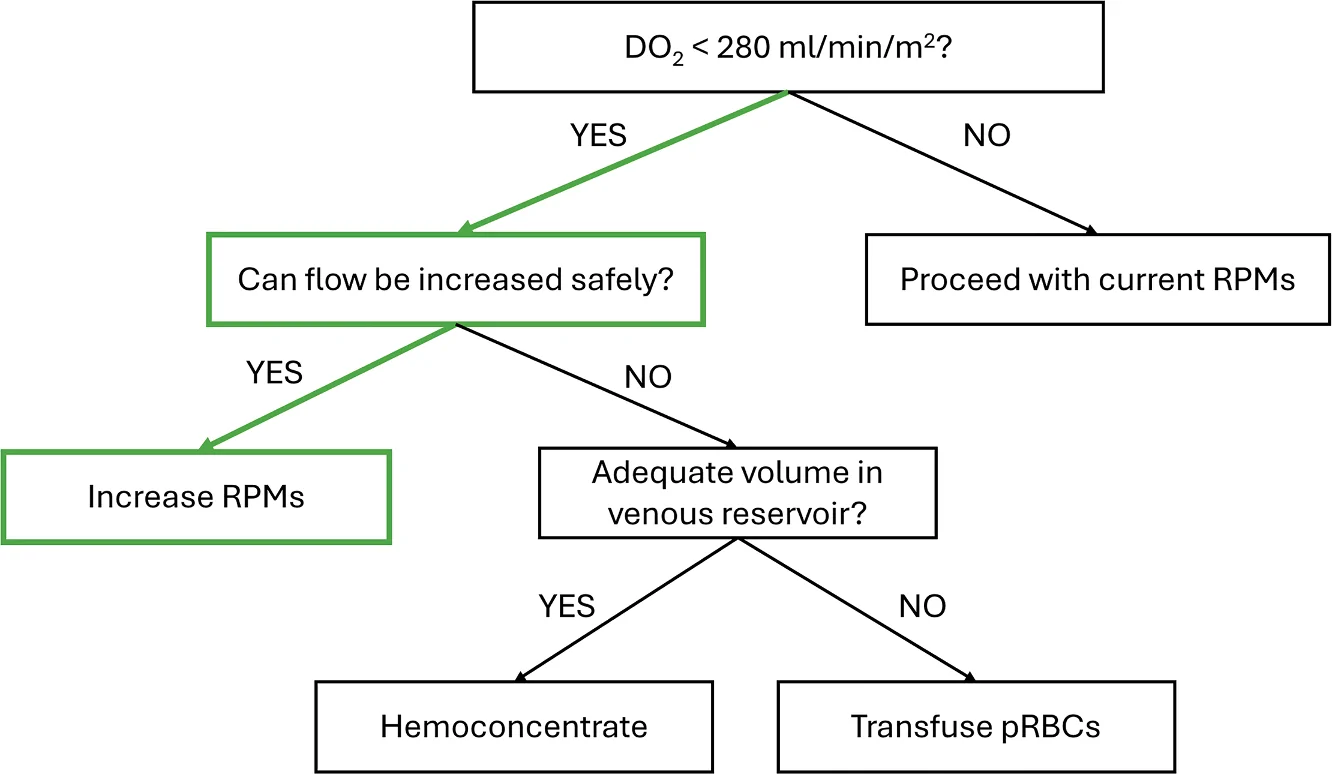
Decision Tree depicting an example explanation with a candidate decision path highlighted. In the figure, DO2 represents Oxygen Delivery, RPMs represent Revolutions per minute, pRBCs represent Packed Red Blood Cells.

Participants also suggested several refinements to improve the usability of decision trees, including: (1) the use of visual cues, such as color coding to highlight relevant decision paths (as shown in Figs. 3), (2) supporting more complex branching logic beyond binary decisions, and (3) enabling access to explanations both during and after procedures for review.

##### Key Takeaways:

Among the evaluated prototype concepts, participants preferred explanation interfaces that used interpretable structures, visual cues, and support for both intraoperative review and retrospective verification.

#### 4.2.3. Live feed

Participants highlighted the importance of maintaining awareness of activities across surgical teams. The concept of a consolidated event feed with actions from all members of the team, including surgery, anesthesia, nursing, and perfusion, was viewed as beneficial, and perfusionists maintained that such information should be available both during and after surgery:

- “It’d be a lot easier to know where they are at in the case, whether it’s for preparing for something or knowing.”
- “If you could figure out how to accurately dictate what’s happening at the surgical field onto something like this. That would be very helpful as a debriefing tool.”

##### Key Takeaways:

**Fig. 4.**
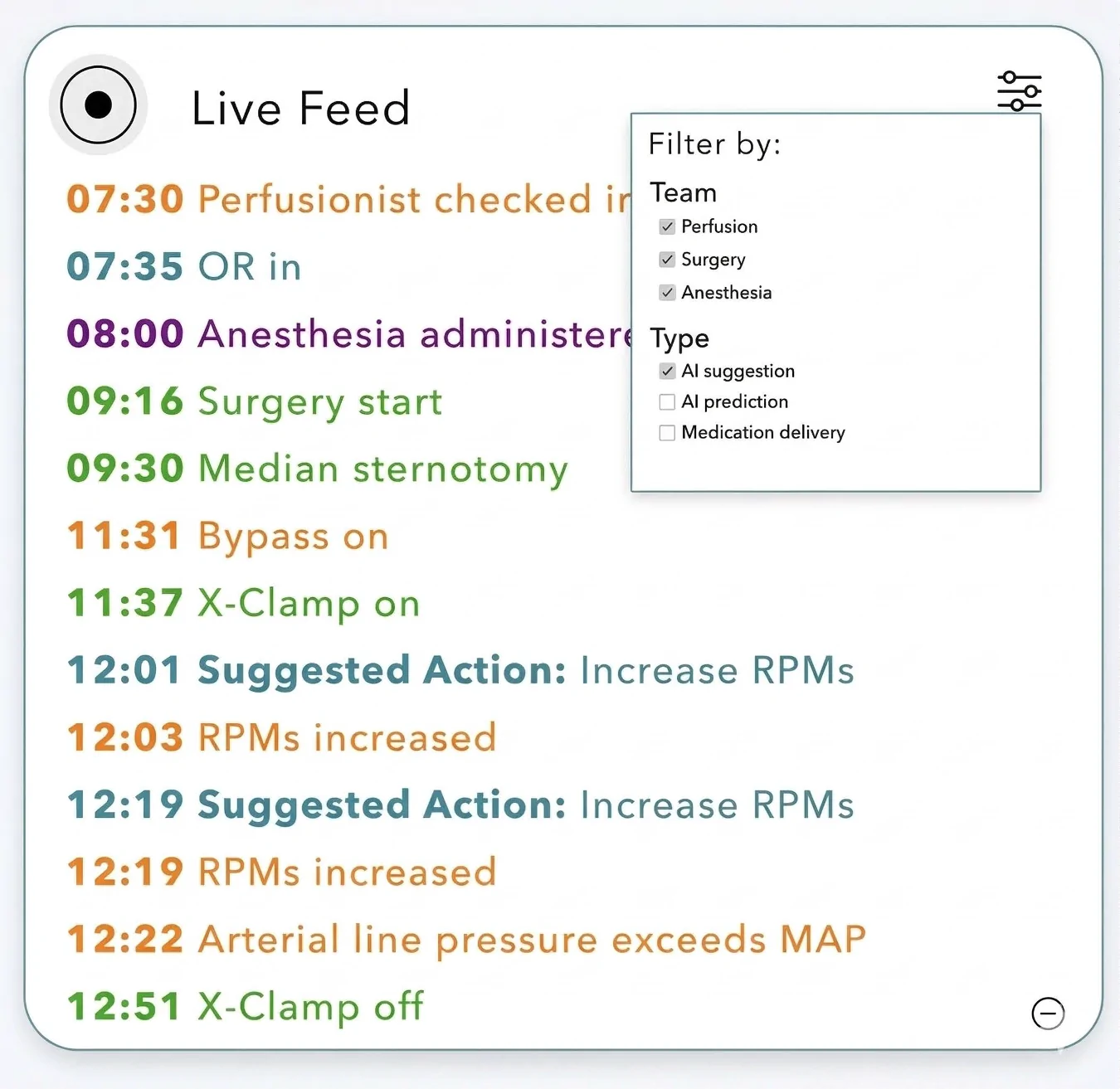
Live feed function. In the figure, MAP represents Mean Arterial Pressure, X-Clamp represents Cross Clamp, AI represents Artificial Intelligence, OR represents Operating Room.

Participants expressed interest in a consolidated display of multi-team information to support intraoperative awareness and postoperative review. Such functionality could potentially be enabled through multimodal sensing approaches, including the integration of intraoperative data streams (e.g., surgical video tracking clinician movements), which have been shown to support continuous capture and analysis of OR activities. (Dias et al., 2023) Further, to enhance usability, participants suggested that the consolidated event feed should support color-coding and filtering based on team and event type, as shown in Fig. 4.

#### 4.2.4. Patient information

**Fig. 5.**
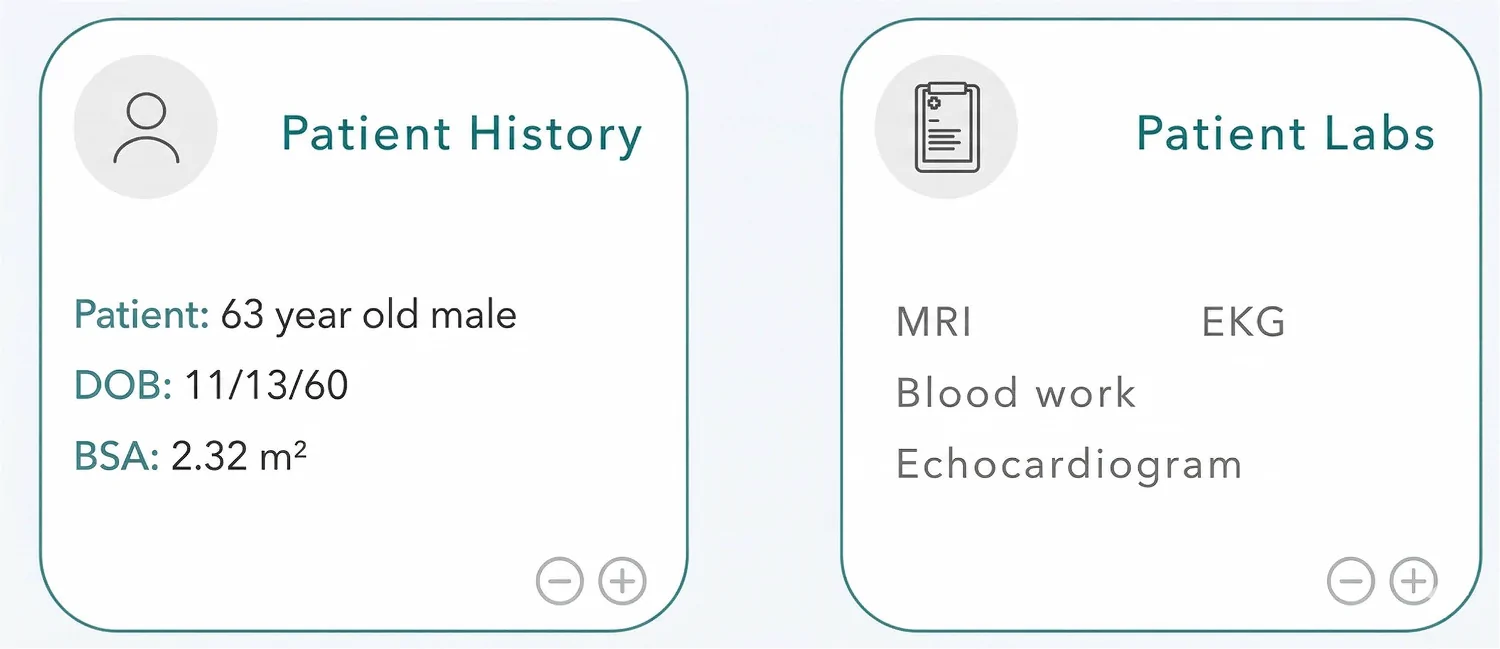
Patient history and labs display. In the figure, DOB represents Date of Birth, BSA represents Body Surface Area, MRI represents Magnetic Resonance Imaging, EKG represents Electrocardiogram.

Participants identified key patient information required for decision-making, including comorbidities, surgical history, medications, allergies, and laboratory values. Participants also emphasized the importance of accessing both historical and day-of patient labs. The relevant patient information identified by perfusionists is depicted on the prototype interface in Fig. 5.

##### Key Takeaways:

Interfaces should support consolidated access to patient information and present relevant data consistent with the clinical context.

### 4.3. Phase 3: usability testing feedback

In Phase 3, we conducted usability testing of a high-fidelity interactive prototype developed based on findings from Phases 1 and 2. We report task performance results and qualitative participant feedback.

#### 4.3.1. Task performance

The overall task completion rate was **84%**, based on independent completion of predefined tasks. The most common difficulties occurred when participants filtered the live event feed and switched between surgery and review modes. These results suggest that most prototype functions were generally accessible to participants, while specific interaction elements may require further refinement.

#### 4.3.2. Participant feedback using the prototype

**Fig. 6.**
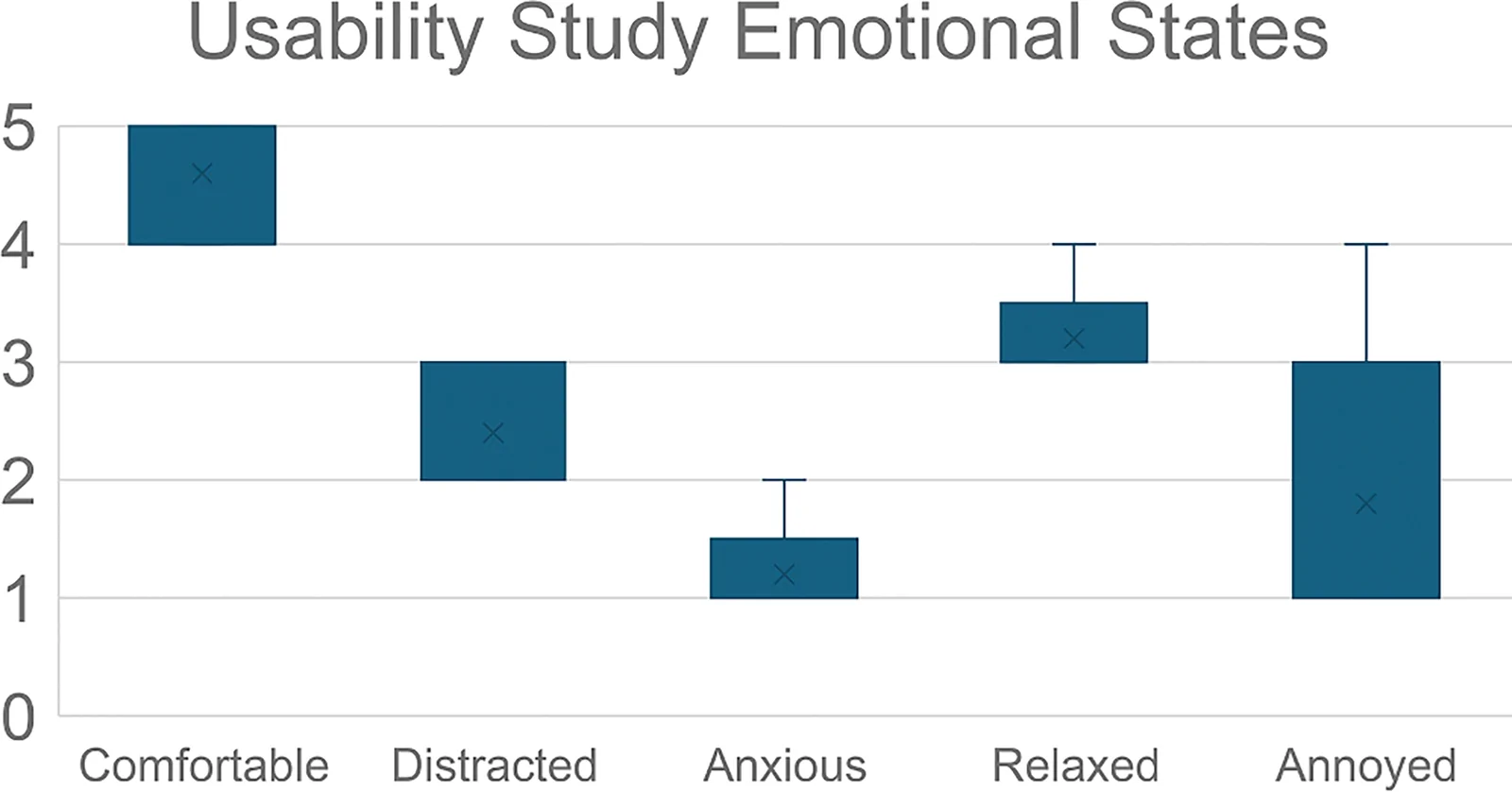
Emotional state data.

Participants reported generally positive perceptions of the prototype interface. Self-reported responses indicated that participants were more likely to feel comfortable and relaxed and less likely to feel distracted, anxious, or annoyed when using the prototype (as shown in Fig. 6).

Qualitative feedback suggested that participants perceived the interface as a potentially useful addition to perfusion practice, particularly for organizing information and presenting decision-support content. However, participants mentioned that they did not want to rely on the output of the AI agent without their own reasoning. Participants appreciated having color-coded live documentation of activities happening in the surgical room available during surgery and debriefs. Participants also noted that decision-making practices varied across institutions and levels of experience and expressed interest in customizable thresholds, alert settings, and information prioritization. Moreover, participants expressed interest in integrating advanced AI technologies, such as computer vision that could capture environmental information during surgery to increase the AI’s awareness and provide contextually relevant suggestions.

### 4.4. Summary of findings

Across all phases, three recurring findings emerged:

1. Decision support systems were perceived as supplementary tools, with participants emphasizing the importance of maintaining clinician control.
2. Interpretability of system outputs was important for understanding system reasoning, with structured formats such as decision trees preferred over the other explanation formats evaluated in the study.
3. Alignment with clinical workflows was considered critical, including compatibility with existing monitoring practices and improved access to relevant patient information.

## 5. Discussion

This study presents a human-centered, participatory design approach to understanding how CDSS may be designed to support perfusionists during cardiopulmonary bypass. Across three phases, we identified user needs, elicited design preferences, and evaluated a prototype interface, contributing insights into the design of decision support systems for a safety-critical clinical domain.

Notably, this work does not evaluate AI system performance or human interaction with operational AI models. Instead, we focus on studying the **design requirements for future intraoperative CDSS** in cardiac perfusion without the confounding effects of model behavior. Such an approach can be crucial in safety-critical domains, where premature integration of AI models without adequate consideration for human factors and clinical workflows can introduce risks related to usability, trust, and patient safety. By first establishing design requirements grounded in clinical practice, this work provides a foundation for the subsequent development and evaluation of AI-enabled CDSS models that are aligned with perfusionists’ needs and clinical workflows.

In this section, we discuss the key findings, iterative improvements in designing the interface, limitations of our findings, and avenues for future work.

### 5.1. Design requirements for future AI-enabled CDSS in perfusion practice

A central finding of this study is that perfusionists were generally receptive to the concept of integrating AI-enabled CDSS into perfusion practice and largely viewed the CDSS as **supplementary tools** to support decision-making, with clinicians retaining the final decision authority. Participants described several workflow demands that CDSS should accommodate, including continuous monitoring of patient vitals, blood-gas values, pump pressure, time on pump, and medication timing; responding to alarms and changes in patient condition; communicating perfusion status to the surgical team; and documenting events during and after procedures. These workflow demands may become more difficult during long cases, when fatigue, competing alarms, and OR activity place additional demands on attention.

Communication was also identified as an important workflow challenge. Noise in the OR, interruptions, and the need to coordinate information across perfusion, surgery, anesthesia, and nursing can make it difficult to maintain a shared understanding of patient and procedural status. Future systems should therefore support clear, timely communication of perfusion-relevant information without adding unnecessary alerts or distracting clinicians from ongoing monitoring. Consolidated displays and timestamped event feeds may support awareness of changes across the operative team, although the effects of such displays on situation awareness were not directly evaluated in this study.

The findings therefore suggest that future CDSS for perfusion should consolidate relevant patient and perfusion information, provide timestamped access to intraoperative events, distinguish alerts and recommendations by urgency, and preserve clinician control by allowing perfusionists to acknowledge, question, reject, or review recommendations. Systems should also accommodate variation across patients, procedures, and institutional practices and provide mechanisms for reviewing recommendations and actions after a case to support debriefing and accountability.

However, several of the design requirements identified above, including information consolidation, reminders, event tracking, and prioritized alerts, could be implemented in a conventional CDSS without AI. AI-enabled functionality would be most relevant when the system adapts recommendations to continuously changing patient and bypass data, communicates uncertainty or the basis for a recommendation, prioritizes information according to clinical context, or adjusts the timing and presentation of support based on estimates of the clinician’s situation awareness or attentional state. (Dias et al., 2022) Such capabilities could potentially help reduce poorly timed or disruptive interventions and support appropriate reliance on automated recommendations.

More broadly, a work-system perspective is needed to examine how future AI-enabled CDSS interact with monitoring, documentation, communication, and supervisory-control activities across the different teams in the OR. Ergonomic analysis can help identify information fragmentation, divided attention, alarm and communication burden, error-recovery needs, and opportunities for postoperative review and debriefing. The requirements identified in this study are intended to support situation awareness, workload management, appropriate reliance, and accountability, although these outcomes were not directly evaluated.

### 5.2. Role of interpretability in CDSS

Among the three explanation formats evaluated, participants consistently preferred decision tree-based explanations due to their structured and interpretable format. These findings contribute to ongoing broader discussions in XAI, where interpretability is often considered a critical factor for improving user understanding. (Rudin, 2019) However, the present findings simply reflect the preferences of participants among the specific explanation formats evaluated and further research is needed to establish whether decision trees improve understanding, appropriate reliance, or clinical performance.

#### 5.2.1. Design implications

Based on our discussions with the participants from the prototype-feedback session (Phase 2), we implemented the following changes in the user interface prior to usability testing:

- User Interface Terminology: The option “Different action” was changed to be called “Alternative action.” The word alternative was more accurate to the action being done.
- Chosen Explanation Format: Decision trees were chosen as the explanation format for usability testing in Phase 3 as participants preferred them over probability-based and counterfactual explanations.
- Additional Visual Cues: Color coding was incorporated into the decision-tree visualization to highlight the presented decision path, enabling users to track the path with ease.
- Intraoperative and Postoperative accessibility: The prototype enabled users to access decision-tree explanations both during and after the simulated procedure.

### 5.3. Interface design to support situational awareness in perfusionist workflows

Participants emphasized the importance of accessing consolidated information about patient data and surgical team activities. These findings identify information-display needs that could be relevant to supporting **situational awareness**, which is critical in dynamic, high-risk environments such as the OR. (Endsley & Jones, 2025) The participants responded positively to the concept of a consolidated event feed with actions from all surgical team members and maintained that such information should be available during intraoperative and postoperative procedures.

#### 5.3.1. Design implications

Based on our discussions with the participants from the prototype-feedback session (Phase 2), we additionally implemented the following changes:

- Clinical teams: The event feed included information concerning surgery, anesthesia, nursing, and perfusion activities.
- Color coding and filtering: Information was color-coded by team and could be filtered according to team and event type, as shown in Fig. 4.
- Interface Placement: The event-feed display was positioned next to the patient vitals monitor, as that was the monitor that most perfusionists were accustomed to seeing in the OR.
- Patient History: The interface included comorbidities, surgical history, medications, and allergies identified by participants as relevant.
- Patient Labs: The interface provided access to historical and day-of-surgery labs.

### 5.4. Feedback from usability testing

The feedback from the final study phase identified additional interface refinements for future work regarding patient information visualization and interaction with decision-support recommendations.

#### 5.4.1. Patient history and labs

- The patient history and patient labs sections of the interface include a tile preview and full-page view. Future versions could allow users to customize tiles or dynamically prioritize information according to clinical context.
- Perfusionists expressed interest in integrating intraoperative labs into the interface.
- A critical next step is to examine the technical feasibility and usability of connecting the prototype to existing electronic medical record (EMR) systems to ensure seamless access to patient data.

#### 5.4.2. Simulated AI agent

- Much of the communication from the conceptual AI Agent to the perfusionist is done using text, which we propose to make more visual and intuitive in future work.
- Participants noted that decision-making practices vary according to institutional standards and professional experience. Future systems could therefore explore customizable thresholds and alert settings.
- Perfusionists also expressed interest in options to hide or dismiss suggestions, trigger a manual review of an AI suggestion for quality assurance, or even provide feedback so that the future AI-enabled CDSS could automatically learn from perfusionist responses and adapt. However, whether such feedback should be used for automatic adaptation would require careful technical and clinical validation.

### 5.5. Limitations

This study has several limitations. With only eight participants recruited through convenience sampling, the findings may not fully represent the diversity of perfusionists, institutions, and clinical practices. Given the exploratory and qualitative aims of the study, this sample size is consistent with prior research indicating that recurring patterns can be identified from relatively small samples, particularly in specialized populations. (Guest et al., 2006) However, we did not conduct a formal saturation analysis, and the findings should therefore be interpreted as context-specific design insights. Future studies should recruit a larger and more diverse sample across institutions and prospectively assess saturation to determine appropriate sample size. Further, we relied on voluntary participation through professional networks, which may have introduced a potential selection bias toward individuals with an intrinsic interest in research or a favorable disposition toward AI-enabled technologies. However, we note that this is an exploratory study to inform the design of future CDSS to be integrated into intraoperative patient care.

Next, our usability testing was conducted with the prototype on an iPad, and only patient vitals at pre-defined timestamps were displayed alongside the presented recommendations. Additional information regarding the patient was provided verbally by the experimenter. This differs from actual OR practice, in which perfusionists must obtain such information from multiple monitors, records, and team members.

For the purpose of this study, the AI interventions were manually created by expert perfusionists and surgeons on the research team based on historical surgical data. Accordingly, the study did not evaluate model accuracy, reliability, latency, or behavior under real clinical conditions. We also did not assess how clinicians calibrate trust, rely on or disregard recommendations, allocate responsibility, or supervise an operational AI system when recommendations are uncertain or incorrect. These questions remain important directions for future evaluation of AI-enabled CDSS in safety-critical settings.

In future clinical implementation, an ML model could be trained to generate candidate interventions using synchronized intraoperative time-series data, such as arterial pressure, pump flow, patient temperature, hematocrit, and oxygen-delivery measures. Such data could be obtained from the sensors on the heart–lung machine, physiological monitors, blood-gas analyzers, and electronic health records, with timestamped perfusionist actions serving as training labels. (Natarajan et al., 2025) Future work will involve developing and evaluating how ML models generate recommendations and adapt based on user feedback and estimated user attention state in real clinical settings.

Finally, much of the evaluation relied on self-reported feedback and an exploratory survey of anticipated emotional responses. Future evaluations should incorporate validated measures of usability (Bangor et al., 2008) and consider assessing behavioral and physiological metrics from the perfusionist, such as gaze (Brunyé et al., 2019) (to assess attentiveness), and heart rate variability (Solhjoo et al., 2019) (for cognitive load).

#### 5.5.1. Usability and evaluation limitations

Usability testing demonstrated that participants were generally able to interact with the prototype and reported positive perceptions of usability. However, consistent with prior work, usability evaluations alone are insufficient to fully assess human-AI interaction in safety-critical environments. (Rayo, 2017; Morey et al., 2023; Morey et al., 2025) In particular, this study does not capture several aspects of real-world deployment: (1) user interaction with deployed AI models, including responses to incorrect recommendations (2) how user trust and reliance on CDSS evolves over time, (3) user behavior under varying levels of system reliability, or (4) how an AI-enabled CDSS would function under real clinical conditions. Additionally, participant responses reflect anticipated use of AI-enabled CDSS, rather than acting under real clinical settings. Therefore, the responses may not reflect actual decision making in practice. (Nisbett & Wilson, 1977) In addition, observed usability errors were not analyzed using a formal failure-analysis framework. Future work should examine user behavioral responses with operational AI models, varying system reliability, apply structured methods such as interaction-failure taxonomies to derive more specific design guidelines, and consider longitudinal user interaction in clinical settings.

## 6. Conclusion

Through exploratory interviews, prototype-feedback sessions, and usability testing session, we gathered design requirements directly from our target users – cardiac perfusionists – and tailored the design of a CDSS prototype to suit their workflow requirements and interface preferences. Rather than evaluating a deployed AI system, this work establishes foundational design requirements and interaction patterns for AI-enabled CDSS in cardiac perfusion. Our findings suggest that perfusionists were generally receptive to the concept of using an AI-enabled CDSS, provided that such systems preserve clinician control and present relevant information and explanations.

### 6.1. Design guidelines

Based on our discussions with the perfusionists across a three-phase multiple-method qualitative study, we propose the following design guidelines for developing future AI-enabled CDSS for perfusion:

- Design for explainability: Use interpretable formats for providing explanations, such as decision trees with layered detail and appropriate highlighting to support inspection of the presented rationale.
- Align with perfusion workflows: Present relevant patient and surgical-team information in a manner compatible with perfusionists’ monitoring practices and support both intraoperative access and postoperative review.
- Enable personalization: Allow customization of thresholds, alerts, and explanation detail to account for variations among individual preferences and institutional practices.
- Preserve Clinician Control: Provide options to accept, reject, dismiss, or request explanations for recommendations while maintaining the primacy of perfusionists’ professional judgment.

Table 2 also summarizes the key findings from the user study and illustrates how these findings map to proposed design guidelines for future AI-enabled CDSS in cardiac perfusion.

### 6.2. Impact statement

By grounding prospective CDSS design in the experiences of perfusionists, this study advances human factors research in high-stakes intraoperative care. The findings provide actionable requirements and interface preferences that can inform the subsequent development and evaluation of future operational AI-enabled CDSS in clinical practice.

## Figures and Tables

**Table 1:** This Table traces how design requirements identified in earlier phases were incorporated in designing prototypes for subsequent phases in the user study.

Identified Design Requirements	Source	Corresponding Prototype Feature or Revision	Phase Incorporated

Review patient health status, medical history, risk factors, medications, and laboratory information.	Phase 1 user stories 1–2	Patient-information display providing access to demographic information, medical history, medications, and laboratory results.	Phase 2 wireframe; retained in Phase 3
Monitor patient vitals, blood gases, urine status, pump pressure, bypass duration, and medication timing.	Phase 1 user stories 8–14	Integrated display of relevant patient and perfusion information, together with a timestamped event feed and procedural history.	Phase 2 wireframe; retained in Phase 3
Need for non-disruptive presentation of information with different levels of urgency.	Phase 1 user story 15; Phase 1 findings concerning fatigue and alarm burden	Visual differentiation and prioritization of recommendations and alerts.	Considered in Phase 2; refined in Phase 3
View system recommendations while retaining control over how to respond.	Phase 1 user story 16; Phase 2 participant feedback	Decision-support panel with options to acknowledge, reject, request an explanation, or request an alternative action.	Phase 2 wireframe
Explore hypothetical actions and their possible consequences.	Phase 1 user story 17	Counterfactual explanation format and alternative-action exploration.	Evaluated in Phase 2
Need for clearer awareness of activities across the surgical team.	Phase 1 user story 18; Phase 1 communication findings	Consolidated live surgical feed showing events across surgical, anesthesia, nursing, and perfusion teams.	Phase 2 wireframe; retained in Phase 3
Revisit system decisions after surgery.	Phase 1 user story 22	Access to explanations and event information for retrospective review.	Evaluated in Phase 2; retained in Phase 3
Preference for optional, non-intrusive recommendations that preserve clinician control.	Phase 2 participant feedback	Retention of user controls and revision of unclear response options.	Phase 3 prototype
Preference for decision-tree explanations, visual highlighting, and access during and after surgery.	Phase 2 participant feedback	Decision-tree explanation interface with highlighted paths and support for retrospective review.	Phase 3 prototype
Confusion regarding the distinction between rejecting an action and requesting a different action.	Phase 2 participant feedback	Revision of response-option wording and interaction design.	Phase 3 prototype

**Table 2:** Traceability of study findings to proposed design guidelines for future AI-enabled CDSS in Cardiac Perfusion.

Design Guideline	Key Supporting Findings	Source	Perfusion-Specific Implication

Design for explainability	Participants wanted to inspect recommendation rationale; preferred decision trees over probability and counterfactual formats; requested a highlighted decision path for the tree format; and requested access during and after procedures.	Phase 2, Sections 4.2.1–4.2.2	Provide concise, visually highlighted explanations that can be interpreted quickly during CPB and retrospectively after the case.
Align with perfusion workflows	Participants described continuous monitoring of vitals, blood gases, pump pressure, bypass duration, and medication timing, and requested consolidated patient data and cross-team information.	Phase 1 user stories 1–2, 8–14; Phase 1, Section 4.1; Phase 2, Sections 4.2.3–4.2.4	Consolidate perfusion-relevant data and team events without disrupting ongoing monitoring.
Enable personalization	Participants noted variations across institutions and experience levels and requested customizable thresholds, alerts, and information prioritization.	Phase 3 usability feedback; Sections 5.4.1–5.4.2	Allow displays and thresholds to be adapted to local protocols, case context, and user preferences.
Preserve clinician control	Participants viewed decision support as a supplementary tool (“second pair of eyes”) and requested options to accept, reject, dismiss, request explanations, or seek manual review.	Phase 1, Section 4.1; Phase 2, Section 4.2.1; Phase 3, Section 4.3.2	Keep recommendations advisory and provide clear controls for responding to or reviewing them.

*Note.* AI = artificial intelligence; CPB = cardiopulmonary bypass; CDSS = clinical decision support system.
